# Supplementary material for: Microbiota-Associated HAF-EVs Regulate Monocytes by Triggering or Inhibiting Inflammasome Activation
Source: Int J Mol Sci. 2023 Jan 28;24(3):2527. doi: 10.3390/ijms24032527 (PMC9916438; doi:10.3390/ijms24032527)
Supplement: Supplementary file 1 [file ijms-24-02527-s001.zip › supplemental figures.pdf]

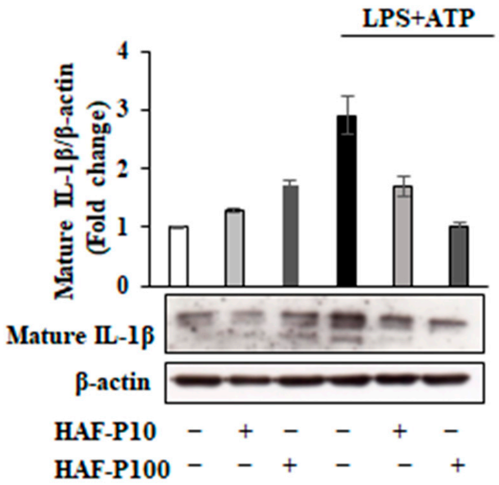

Figure S1. HAF-EV modulate IL1β expression. THP-1 cells were pre-treated with HAF-EVs (P10 or P100) for 1 h, and subsequently primed with 10μg/mL LPS for 20 min and then activated with 5 mM ATP for 40 min (LPS + ATP). Cell lysates were immunoblotted for IL-1β.

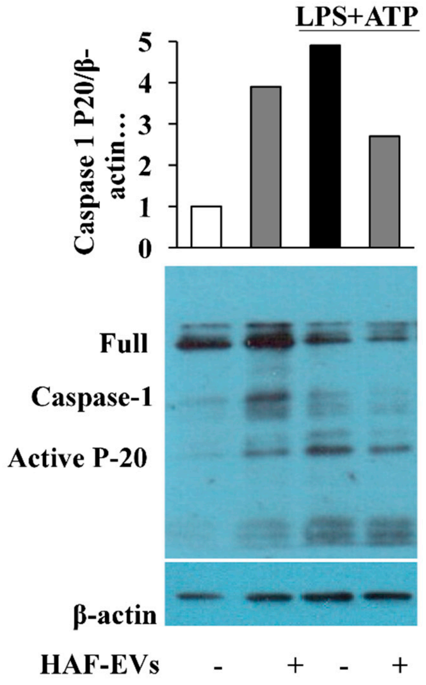

Figure S2. Regulation of the Inflammasome by HAF-EVs in Human monocytes. The cells were pre-treated with 100 μg/mL of HAF-EVs for 1 h, and subsequently primed with 10 μg/mL LPS for 20 min and then activated with 5 mM ATP for 40 min (LPS + ATP). Cell lysates were immunob-lotted for Caspase-1. β-actin was used as a loading control. Histograms represent densitometric quantification.

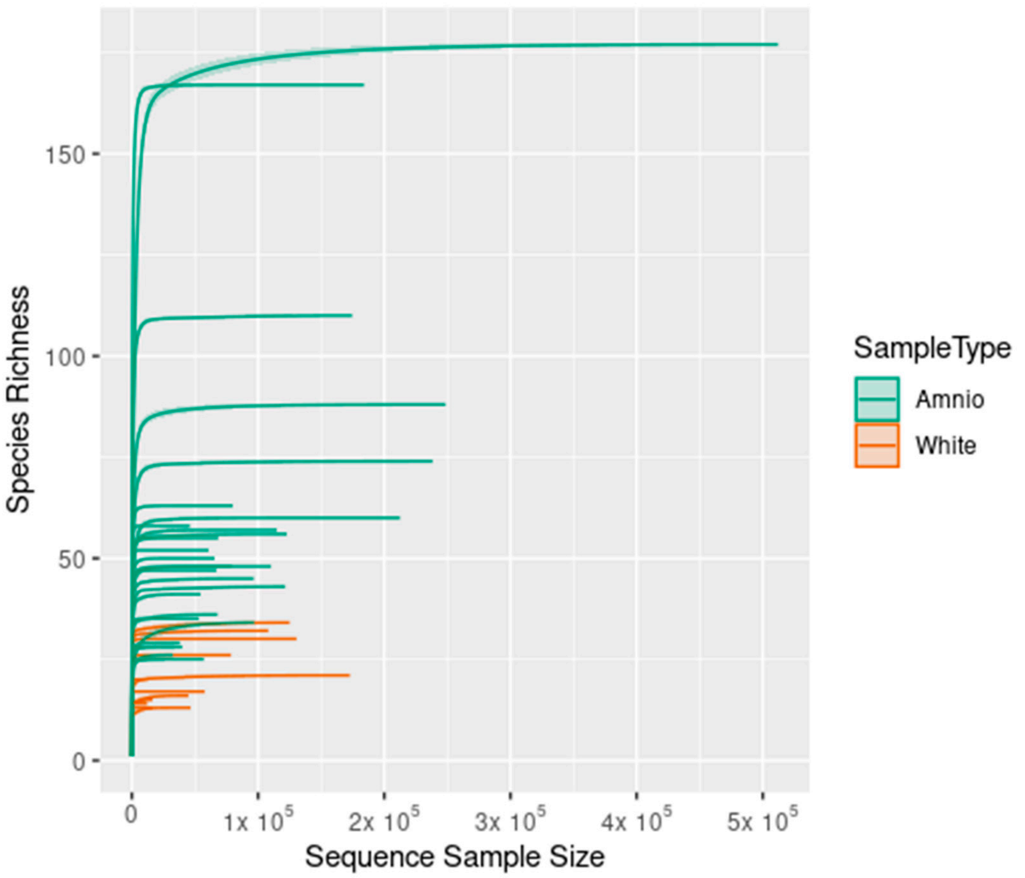

Figure S3. Rarefaction curve of all samples. Rarefaction Curve shows the behavior of the species richness of each sequenced sample versus the corresponding sequencing depth and emphasizes that all samples have been sufficiently sequenced with a good sequencing quality since richness has a flat behavior. Minimum and maximum sampling depth of samples are 12,212 and 51,2071, respectively (average value equal to 96,111).

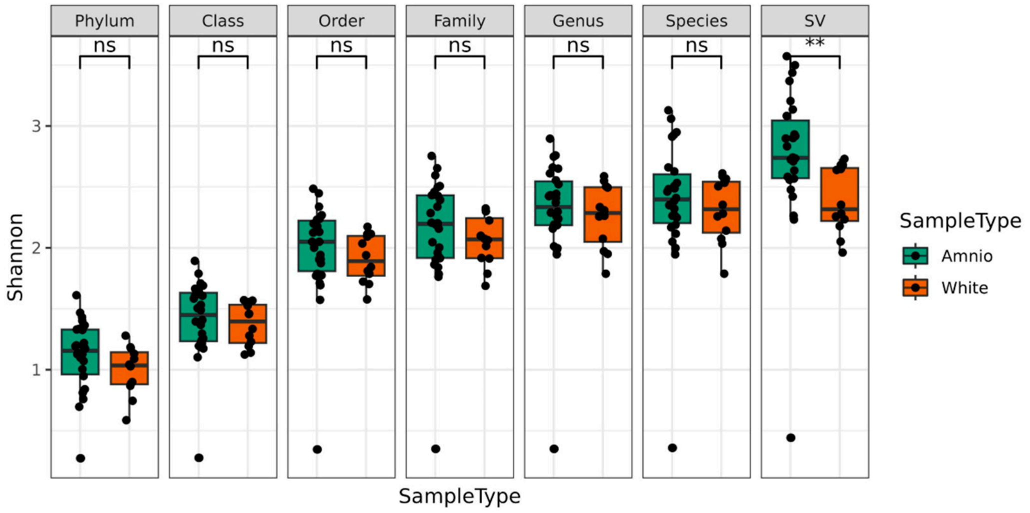

Figure S4. Shannon alpha diversity. Shannon alpha index evaluated in each group (green: amni-otic liquid; red: blanks) at each taxonomic level as indicated by the corresponding label. Com-parison between groups (KruskallWalli test) is also reported. Ns is not significant, ** $p < 0.01$.

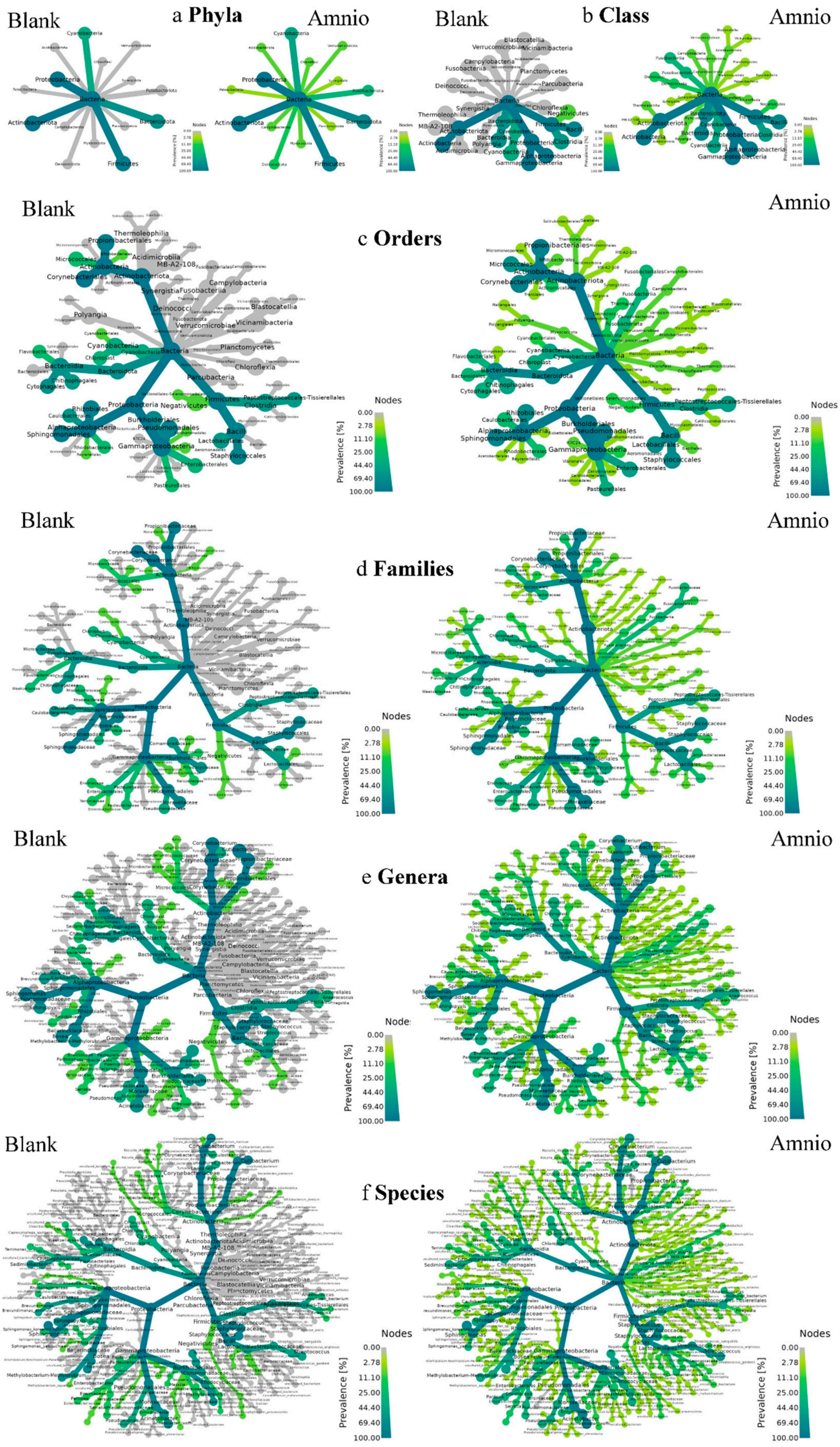

Figure S5. Heat tree of sequence variant (SV) in Blank.

Variant in Blank

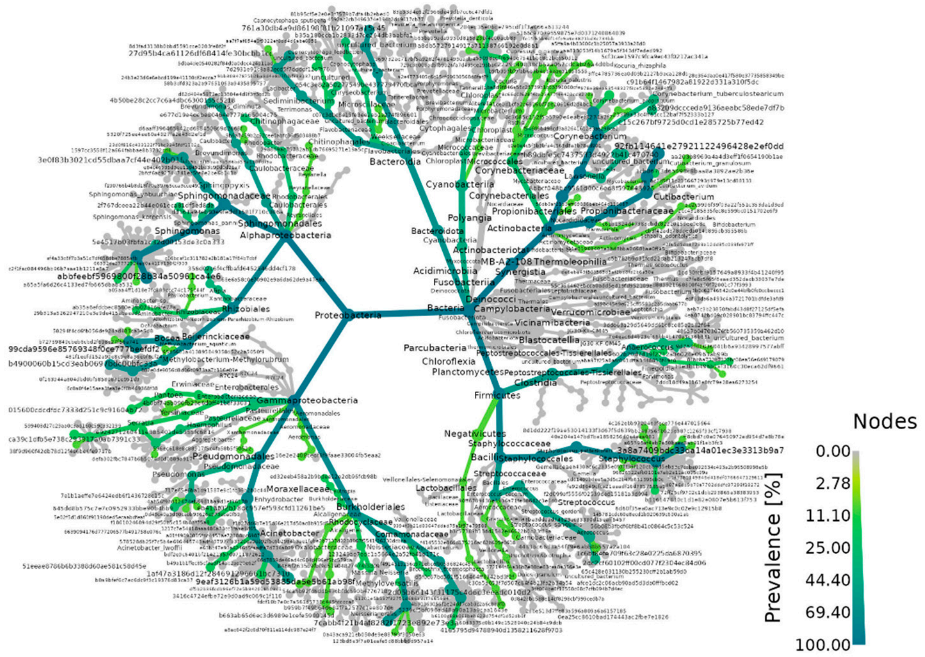

Figure S6. Heat tree. Heat tree of samples and white grouped into taxonomic level. The preva-lence (%) is expressed as the percentage value of samples containing the specific taxa. A pseu-do-count of 1 ×10−4% was used on percentage values.

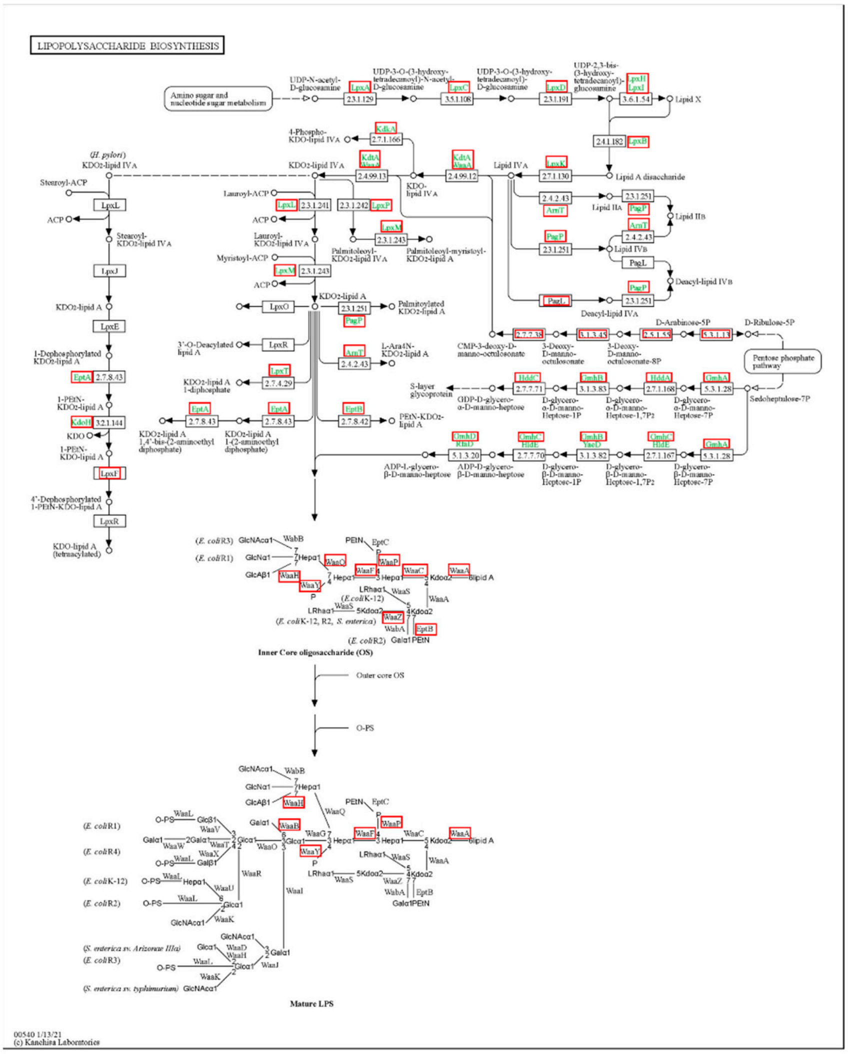

Figure S7. Biosynthes of Lipolysaccharide. Modified scheme of the pathway ko00540 Lipopoly-saccharide biosynthesis (https://www.genome.jp/pathway/map00540) from KEGG ORTHOLOGY. Gene names of enzymes involved in the path-way are defined in green; genes predicted by func-tional analysis are marked by a red box.
