# Supplementary material for: Microbiota-Associated HAF-EVs Regulate Monocytes by Triggering or Inhibiting Inflammasome Activation
Source: Int J Mol Sci. 2023 Jan 28;24(3):2527. doi: 10.3390/ijms24032527 (PMC9916438; doi:10.3390/ijms24032527)
Supplement: Supplementary file 1 [file ijms-24-02527-s001.zip › Table S1.pdf]

Table S1. Proteins identified in HAF-P10 and HAF-P100

HAF-P10		HAF-P100	
**Gene name**	**Protein full name**	**Gene name**	**Protein full name**
ANXA1	Annexin A1	ANXA1	Annexin A1
ANXA13	Annexin A13	ANXA13	Annexin A13
ANXA2	Annexin A2	ANXA2	Annexin A2
ANXA4	Annexin A4	ANXA4	Annexin A4
ANXA5	Annexin A5	ORM1	Alpha-1-acid glycoprotein
ORM1	Alpha-1-acid glycoprotein	ANPEP	Aminopeptidase
GAPDH	Glyceraldehyde -3 phosphate dehydrogenase	APOA1	Apolipoprotein A-I
IGKC	Immonuglobulin Kappa Costant	B2M	Beta-2-microglobulin
AMBP	Protein AMBP	CP	Ceruloplasmin
SMC5	Structural maintenance of chromosomes protein	COL6A1	Collagen alpha-1
VTN	Vitronectin	DPP4	dipeptidyl peptidase
ACTB	Actin cytoplasmic 1	FN1	Fibronectin
SERPINA1	Alpha 1-antitrypsin	HBG1	Hemoglobin subunit gamma-1
CLU	Clusterin	IGLV3-21	Immunoglobulin lambda variable
ELAVL1	Elav –Like protein 1	SI	Sucrase Isomaltase
HBB	Hemoglobin sub unit beta	YWHAB	14-3-3 protein beta/alpha
HBA1	Hemoglobin subunit Alpha	YWHAZ	14-3-3 protein zeta/delta
HBB	Hemoglobin subunit Beta	ACTA2	Actin, aortic smooth muscle
HBD	Hemoglobin subunit Delta	ARF1	ADP-ribosylation factor 1
HBG1	Hemoglobin subunit Gamma 1	SERPINA1	Alpha 1-antitrypsin
HBG2	Hemoglobin subunit gamma-2	A1BG	Alpha-1B-glycoprotein
IGKV2D-26	Immonuglobulin kappa variable	ACTN1	Alpha-actinin-1
AMBP	Protein AMBP	ACE2	Angiotensin-converting enzyme2
TF	Serotransferrin	APOA4	Apolipoprotein A-IV
ALB	Serum albumin	APOB	Apolipoprotein B-100
		HSPG2	Basement membrane-specific heparan sulfate proteoglyca core protein
		SLC4A4	bicarbonate cotransporter
		PRG2	Bone marrow proteoglycan
		BPIFA1,	BPI fold-containing family A member 1
		CAH1	Carbonic anhydrase 1
		CLIC1	Chloride intracellular channel protein 1
		CLU	Clusterin
		COL1A1	Collagen alpha-1(I) chain
		COL6A3	Collagen alpha-3(VI)
		C3	Complement C3
		CFB	Complement factor B
		EGLN2	Egl nine homolog 2
		SLC4A4	Electrogenic sodium
		FTL	Ferritin light chain
		FN1	Fibronectin
		GGT1	Glutathione hydrolase 1 proenzyme
		GSTO1	Glutathione S-transferase omega-1
		GOLPH3L	Golgi phosphoprotein 3-like
		GNA11	Guanine nucleotide-binding protein subunit alpha-11
		HBA1	Hemoglobin subunit Alpha
		HBB	Hemoglobin subunit Beta
		HBG1	Hemoglobin subunit Gamma 1
		HBG2	Hemoglobin subunit gamma-2
		H2BC1	Histone H2A type 1-B/E
		FCGBP	IgGFc-binding protein
		IGG1	Immunoglobulin gamma-1
		IGG1	Immunoglobulin gamma-1 heavy chain
		IGHG2	Immunoglobulin heavy constant gamma 2
		IGKC	Immunoglobulin kappa
		IGKC	Immunoglobulin kappa constant
		IGL1	Immunoglobulin lambda-1
		IGL1	Immunoglobulin lambda-1 light chain
		ITIH4	Inter-alpha-trypsin inhibitor heavy chain H4
		MEP1A	Meprin A subunit alpha
		TIMP1	Metalloproteinase inhibitor 1
		MME	Neprilysin
		PRDX1	Peroxiredoxin-1
		PEBP1	Phosphatidylethanolamine-binding protein 1
		SERPINF1	Pigment epithelium-derived factor
		SERPING1	Plasma protease C1 inhibitor
		PLG	Plasminogen
		PRL	Prolactin
		PROM1	Promini-1
		AMBP	Protein AMBP
		FAM3C	Protein FAM3C
